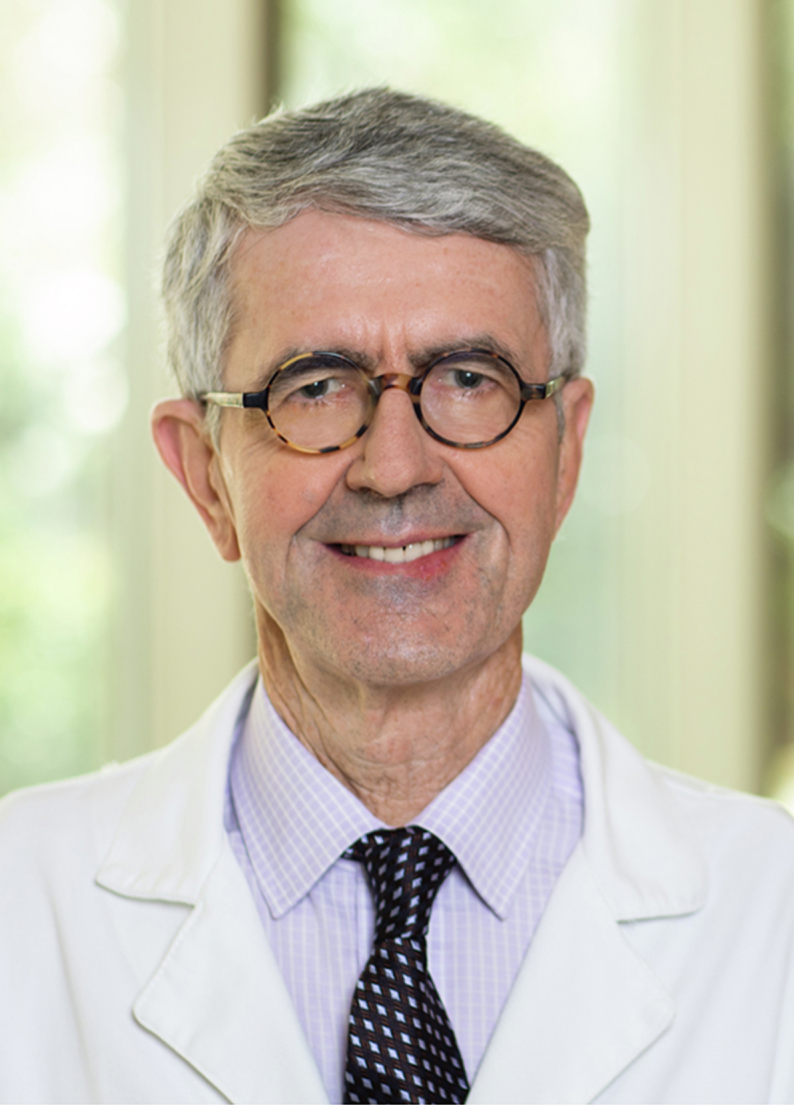# Pearls From the Pros: Hepatic Presentations of Celiac Disease

**DOI:** 10.1016/j.gastha.2023.03.018

**Published:** 2023-03-21

**Authors:** Lawrence S. Friedman, Paul Martin

**Affiliations:** 1 Department of Medicine, Newton-Wellesley Hospital, Newton, Massachusetts; 2 Massachusetts General Hospital, Harvard Medical School, and Tufts University School of Medicine, Boston, Massachusetts; 3 Miller School of Medicine, University of Miami, Miami, Florida

Celiac disease can present with extraintestinal manifestations, including liver disease, with a spectrum ranging from elevated liver biochemistries to advanced cirrhosis. Liver biopsy findings may include variable degrees of steatosis, inflammation, and fibrosis. In one case, we have seen the patient presented with unexplained ascites and features suggestive of Budd-Chiari syndrome. The serum-ascites albumin gradient was 2.3 with a total protein of 0.8 g/dL, and albumin 0.5 g/dL, with an ascitic white blood cell count of 88/mm^3^. Echocardiography showed an ejection fraction of 80%. Transjugular liver biopsy revealed a normal hepatic venous pressure gradient but marked sinusoidal dilatation and congestion with hepatocyte atrophy and focal necrosis suggestive of vascular outlet obstruction (Figure A). Hepatic venography, however, showed no evidence of Budd-Chiari syndrome.

**Figure 1:**
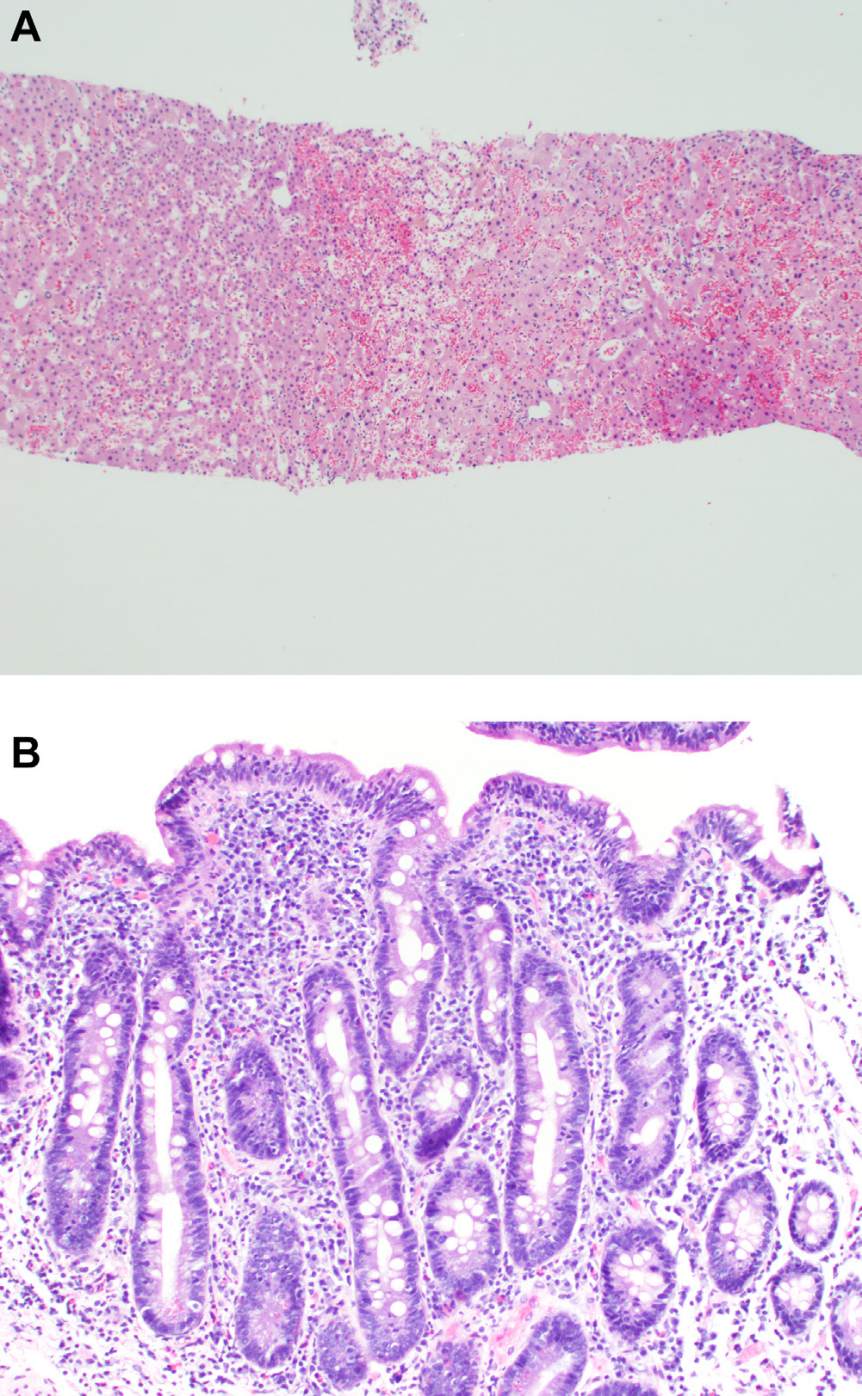

When seen in consultation, pertinent observations included Irish ancestry, a history of occasional diarrhea, short stature, osteoporosis, and an atrophic spleen on computed tomography. An Immunoglobulin A transglutaminase antibody was positive, and a small bowel biopsy confirmed celiac disease (Figure B). On a gluten-free diet, her symptoms resolved, with clinical and laboratory abnormalities returning to normal. She lived another 20 years before dying of primary pulmonary hypertension. Recognition of an unusual hepatic manifestation of celiac disease led to effective management.

**Figure:**
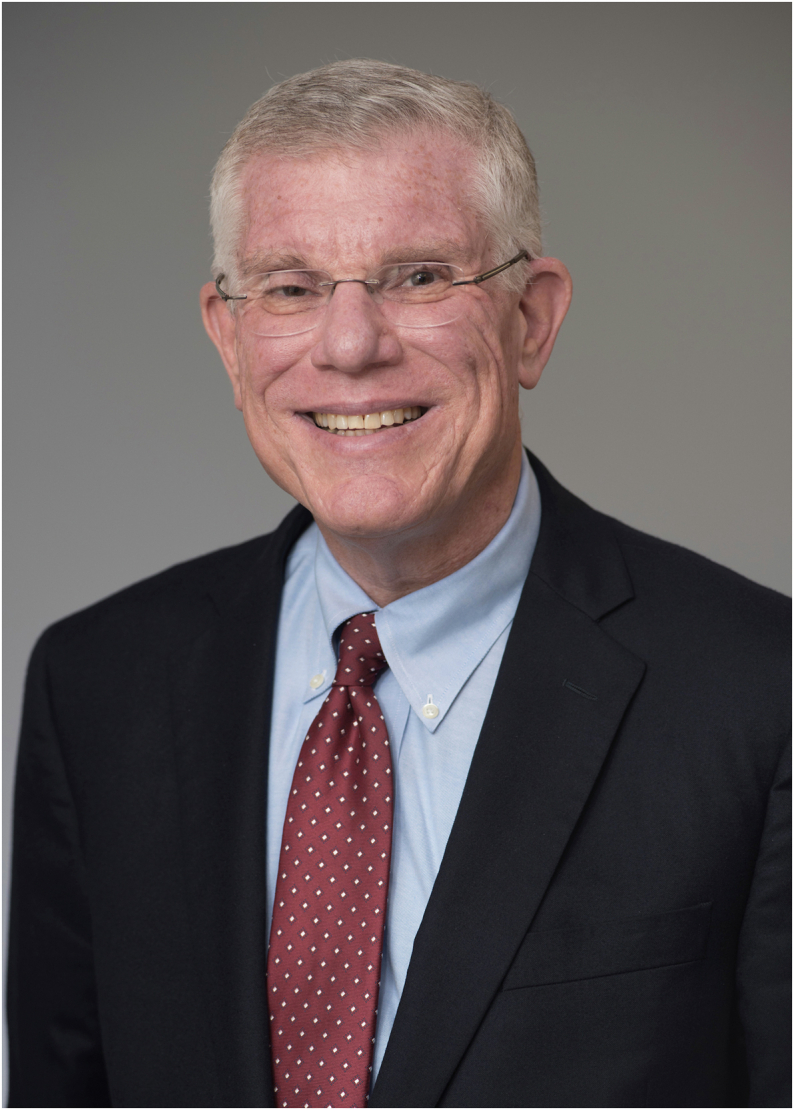

**Figure:**